# An exploration of mediating mechanisms influencing willingness to pay for corporate social responsibility

**DOI:** 10.1038/s41598-025-16901-w

**Published:** 2025-10-02

**Authors:** Sohail Ahmad, Qingyu Zhang, Zaheer Ahmad, Aman Ullah

**Affiliations:** 1 Research Institute of Business Analytics and Supply Chain Management, College of Management, Shenzhen University, 518060 Shenzhen, China; 2 Government College of Management Sciences, Mardan, Pakistan

**Keywords:** CSR, Willingness to pay, Emotional attachment, Consumer happiness, Consumer retention, Service quality, Environmental sciences, Environmental social sciences

## Abstract

Corporate social responsibility is known a significant factor in shaping consumer behavior, particularly in socially conscious and developing economies. However, limited research has observed the psychological mechanisms through which CSR affects consumers’ willingness to pay. The objective of the study is to examine how CSR influences WTP through the mediating roles of consumer happiness, retention and emotional attachment, while also exploring the complementary role of service quality in strengthening these relationships. Drawing on cognitive consistency, stakeholder and perceived value theories, a conceptual framework was developed and tested using data from 350 consumers in Pakistan. The results reveal that CSR indirectly influences WTP through the mediators, but does not exert a significant direct influence. In contrast, service quality shows a significant direct and indirect influence on WTP. The findings propose that emotional and relational factors mediate the relationship of corporate social responsibility and willingness to pay and highlight the importance of integrating corporate social responsibility with high service quality to improve consumer satisfaction and price tolerance. The study offers theoretical and managerial implications for using corporate social responsibility and service quality to foster long-lasting consumer relationships and improve pricing strategies in developing economies.

## Introduction

In today’s fast evolving market landscape, corporate social responsibility (CSR) and service quality have emerged as crucial factors that may influence consumer behavior, especially in shaping consumer’s willingness to pay (WTP). CSR, encompassing a business’s commitment to address economic, social, and environmental concerns, not only advances corporate reputation but also fosters trust and loyalty among consumers^1,2^. By indicating ethical responsibility through CSR activities, businesses indicate their alignment with social values, which has positive impacts on consumer’s perceptions and purchasing decisions^3^. Service quality may serve as an important driver of consumer satisfaction and strengthen long-term relationships and increasing WTP^4^. Existing research has recognized the individual influences of CSR and service quality on consumer behavior^5–9^ there remains a significant gap in understanding how they interact to influence WTP, specifically in the context of developing economies. Existing studies often focus on direct influence or mediators such as brand credibility^10^ and brand equity^11^ but they often overlook the emotional and psychological mechanisms such as consumer happiness, retention, and emotional attachment that mediate these relationships. The objective of the study is to address this gap by constructing an integrated framework that inspects the combined influence of CSR and service quality on WTP, mediated by consumer happiness, retention, and emotional attachment with a focus on emerging market like Pakistan.

The research problem lies in the limited understanding of combined influence of CSR and service quality in shaping consumer’s WTP, particularly through psychological and emotional mediators. While CSR enhances trust, loyalty, and corporate image^7,12^ and service quality drives satisfaction and retention^13,14^ the interplay between these factors and their combined influence on WTP remains underexplored. Most studies treat CSR and service quality as independent variables, failing to account for their synergistic impact on consumer decision-making^15,16^. Furthermore, emotional mediators such as consumer happiness, retention, and emotional attachment, which are critical in linking CSR and service quality to WTP, have received insufficient attention^8,17,18^. This gap is particularly pronounced in developing economies like Pakistan, where CSR and environmental awareness is growing^19,20^ and service quality varies significantly across sectors. Recent literature highlight that Pakistani consumers increasingly value CSR practices, particularly those emphasizing social welfare and ethical conduct, as key determinants of brand evaluation and purchase intention^21,22^. However, the lack of an inclusive framework integrating CSR, service quality and emotional mediators restricts the capability of businesses to use these factors to justify premium pricing.

The significance of this problem is heightened in developing markets, where consumer awareness of ethical business practices is rising. CSR initiatives are increasingly shaping consumer preferences and loyalty in Pakistan^23^. However, businesses often lack clear insights into how CSR and service quality can combined improve WTP, particularly when mediated by psychological and emotional factors. Previous research indicates that CSR can positively influence consumer satisfaction and perceptions of quality, thereby increasing WTP^11,24^ but these effects are not universal and may depend on consumer demographics, such as age and income, as well as cultural and psychological factors^25–28^. For example, younger and wealthier consumers are more likely to pay premiums for socially responsible products^25^. Additionally, the linkage between CSR awareness and purchase behavior is influenced by consumers’ attitudes toward CSR and emotional connections to brands^29^. Despite these findings, the mediating roles of consumer happiness, retention, and emotional attachment in the CSR, service quality and WTP relationship remain underexplored, particularly in developing economies.

Consumer happiness, which is the emotional feeling of contentment and pleasure from buying goods or services, is key in connecting CSR^8^service quality^17^and willingness to pay. Consumers who are pleased are more inclined to express positive reviews of a business’s CSR efforts and products, making them willing to pay^15,30^. Also, the ability of an organization to keep its consumer base, known as consumer retention, is influenced by both CSR and service quality, which impact consumer’s willingness pay^16,31^. Another important mediator is emotional attachment, which pertains to consumers’ perspectives and feelings of a business’s CSR initiatives. Consumer’s emotional attachment can increase loyalty and support higher prices since they are willing to pay premium for products or services from socially responsible businesses^18,30^.

To address this gap, the study offers an integrative framework based on cognitive consistency, stakeholder, and perceived value theories to examine the joint influence of CSR and service quality on WTP, mediated by consumer happiness, retention, and emotional attachment. Cognitive consistency theory suggests that consumers align their attitudes and behaviors, but it does not fully account for emotional factors like happiness, which are critical in driving WTP^32^. Stakeholder theory emphasizes aligning business practices with stakeholder interests but often overlooks emotional and relational mediators like attachment and retention^33^. Perceived value theory, while focusing on tangible benefits, tends to neglect the intangible benefits of CSR and service quality, such as social value and emotional satisfaction, which enhance WTP^34^. By incorporating these emotional mediators, this study extends these theories to provide a more comprehensive understanding of consumer behavior in response to CSR and service quality.

The objective of this study is to investigate the joint influence of CSR and service quality on consumer WTP, with consumer happiness, retention, and emotional attachment as key mediators, in the context of Pakistan’s fast-growing industrial sector and increasing CSR awareness. An empirical survey of 350 consumers in Pakistan was conducted to test these hypothesized relationships. By examining these mediators, the study provides actionable insights for businesses to tailor their CSR and service quality strategies to strengthen consumer relationships and justify premium pricing. The findings will guide decision-makers in emerging markets, where CSR initiatives require significant investments that can be recouped through enhanced WTP. This research contributes to the literature by filling the gap in understanding the emotional and psychological mechanisms linking CSR, service quality, and WTP, offering a robust framework for consumer decision-making in socially and economically transitional markets.

## Literature review

### Corporate social responsibility

CSR has four basic dimensions: economic, legal, ethical, and philanthropic responsibilities^35^. The overall goal of CSR is to ensure that the social benefits created by such activities foster sustainability and profitability for businesses^36^. A focus on social consciousness can enhance performance, as improved working conditions are apt to maximize employee commitment and performance^37^. Despite the large corpus of literature related to CSR, there are significant gaps in understanding the impacts of certain CSR practices on consumer behavior, specifically on their willingness to pay (WTP) for products or services. Many studies discuss CSR in general terms without specifying the different impacts of different CSR activities on consumer decisions, such as economic, legal, ethical, and philanthropic aspects^38^. Researchers have explored behavioral intention^39,40^ purchase intention^41^consumer awareness^42^ satisfaction^23^ and engagement to understand the impact of CSR on consumer behavior^43^. It would be essential to examine various aspects of CSR for organizations that adopt CSR, as this might enable more effective incorporation of CSR into their practices and foster positive consumer relationships^44^.

### Corporate social responsibility and consumer behavior

In recent years, CSR has emerged as a prominent subject of discussion among businesses and consumers, underscoring the significance of social responsibility in shaping public perception and consumer behavior. CSR, which covers ethical business, environmental sustainability, and societal contributions, influence brand reputation and customer loyalty^45^. According to research, CSR leads to higher consumer trust, brand admiration, and satisfaction, which positively influences the consumer WTP^16^. However, existing literature is inconclusive about the direct link between CSR and WTP. Some research suggest that CSR alone might not be impactful enough to charge a premium without adding other value-driving elements^46^ such as perceived service quality or emotional connection. This gap in perception the precise procedures through which CSR influences WTP presents an opportunity for further investigation. Recent research illuminates the role of emotional and psychological factors in driving consumer’s CSR-focused behavior. For example^8^, conducted a research and determined that, CSR is a catalyst of consumer happiness and happiness in return significantly increases purchase intention. Similarly^47^, found that an emotional attachment is key to converting CSR perceptions into long-term brand loyalty. These studies indicate that the effect of CSR on WTP is not linear but mediated through consumers’ emotions and engagement, yet these mediators have not been adequately explored in the context of CSR and WTP, especially in developing economies like Pakistan.

### Corporate social responsibility in Pakistan

The topic of CSR is new to Pakistan. Many scholars believe that CSR is still in its early phases in Pakistan^48–50^. Some focus on corporate generosity, whereas others consider CSR the state norms and standards, paying wealth through taxes and employee welfare.

In Pakistan, many specialized organizations endorse CSR. An Asian forum on CSR was created by the Asian Foundation of Management, which encouraged CSR projects. This group evaluates and honors CSR actions by Asian firms in environmental preservation, poverty reduction, health, and education. These sorts of corporate endeavors empower CSR execution in Pakistan and can assist with making a steady and prosperous environment at the grass-root level. However, whether or not these high-profile organizations can reach out to the grassroots is a point of contention^51^.

Most businesses operating in evolving economies for instance Pakistan are reactive to the principle of adherence to administration rather than proactive such as local textile manufacturing companies, small scaled retail businesses, domestic food manufacturing units, etc. To illustrate, a large number of textile companies only comply with the requirements of international buyers when it is a strict requirement and fail to adopt sustainable and socially responsible practices in a proactive manner. For example, the food and beverage industry’s SMEs only adopt environmental standards or labor rights initiatives if forced by regulators and other stakeholders to comply. As a result, the government must compel multinational corporations (MNCs) to contribute to society’s well-being, such as health, poverty mitigation, and ecological safeguard, by enacting and imposing strict instructions^50^. In addition to the government, business and nonprofit organizations must take part effectively to promote CSR^50^. As a result, it can be stated that CSR in Pakistan is currently in its early stages. Few multinational corporations practice CSR policies. At the same time, most local businesses are either uninformed of CSR’s advantages or unconcerned about government penalties in the event of non-compliance. To address this, Pakistan needs a sizeable public outreach movement in which all relevant parties should participate.

### Theoretical support

The study constructs an in-depth model that demonstrates the influence of CSR and service quality on WTP through mediators such as consumer happiness, emotional attachment, and retention, based on cognitive consistency, stakeholder, and perceived value theories. Recent work highlights the relevance theories for understanding complex consumer decisions in contexts of CSR. Rendering to cognitive consistency theory, people pursue harmony between their attitudes, beliefs, and actions^52^. Recent studies emphasize its importance in terms of CSR offering relevance demonstrating that positive perception towards CSR of businesses lead to favorable behavioral manifestation like loyalty and advocacy^53^. However, these studies often neglect to incorporate emotional mediators such as happiness and attachment, which may influence and shape WTP. By expanding this theory, this study shows how CSR accommodates consumer values and emotions, establishing willingness to pay premium. The gap in the literature is that cognitive consistency theory has not sufficiently incorporated emotional drivers like consumer happiness and retention as mediators in CSR-WTP relationships, which this study aims to address^54^.

Stakeholder theory argues that organizations should consider the interests of all stakeholders, not just shareholders^55,]^^56^. highlight that firms aligned with stakeholder expectations, especially in terms of CSR, will likely enjoy increased consumer loyalty. Nevertheless, there is limited empirical work that bridges the mediating effects of emotional attachment and retention, which reflect CSR’s influence on consumer retention and WTP. The aim of this research is to bridge this research gap by showing CSR initiatives directed at stakeholders as key drivers of emotional attachment and consumer retention, which ultimately lead to greater economic returns. While stakeholder theory is strong on relational dynamics, it fails to explore the role of emotional attachment as a mediator in these relational constructs. Perceived value theory outlines consumer behavior as a result of the dynamic between perceived benefits and related costs^57,]^^11^. verified that CSR activities lead to a rise in perceived value toward WTP. However, many studies fail to explore the interaction between perceived value, emotional attachment, and consumer satisfaction features indispensable to understanding the relationship between CSR and WTP. Through an extension of perceived value theory, this study provides insights into how social and emotional benefits influence WTP. Through the integration of these components, the study enriches the theoretical base by proposing a more sophisticated understanding of the complex relationship between CSR, service quality, and consumer WTP.

This study overcomes the shortcomings in current theory by incorporating cognitive, affective, and relational forces that occur between consumers and firms an area that has been under addressed by the current literature. In doing so, it adds value to scholarly debate by making contributions to current empirical research in the fields of CSR, service quality, and their effects on WTP. In addition, it offers an in-depth investigation of the mediating processes that drive these relations, thus enabling a deeper understanding of the interplay between these variables. Additionally, it offers evidence-based insights for managers looking to enhance financial performance through socially responsible practices. The psychological harmony described by cognitive consistency theory between consumer values and behaviors show limited recognition for emotional relationships including happiness and attachment. Stakeholder theory concentrates on stakeholder interest often neglects the emotional processes required for consumer retention and establishing emotional attachment. Perceived value theory principally analyzes monetary trade-offs between price benefits and costs but it often neglects to fully assess emotional benefits from CSR involvement. The research incorporated multiple theories to overcome their individual shortcomings while developing a framework that analyzes cognitive and emotional consumer engagement to determine WTP. The theoretical consolidation enhances financial analysis of how CSR and service quality create financial effects by establishing emotional connections with consumers.

## Conceptual framework and hypotheses development

### Corporate social responsibility and willingness to pay

CSR refers to voluntary actions by corporations to incorporate social concerns into decision-making, business practices, and interactions with society so as to promote particular social objectives. Measures that are linked to CSR cover a wide range of social issues such as public health, human rights, environmental conservation, and social security^58,59^. There is proof that CSR can enhance strategic business goals^60^ and financial performance^61^. Based on a vast and expanding body of scientific data, we argue that CSR is the best way for corporations to address important social issues and promote social welfare. The concept of happiness as a company’s responsibility has been evidenced in the CSR literature. It is more reasonable to persuade the business to do something good to enhance social and environmental condition, which would increase the well-being of human beings and the quality of their lives^60^. To not only maintain and develop the objective state of the society but also enrich the subjective perceptions^60^.

Throughout the last few decades, businesses have been working to increase the happiness of their target audiences^62^. Business strategies can be created to enhance consumer happiness and go beyond hedonic delight and flimsy service offerings^63^. Hence, CSR may add value and improve any consumer-focused business strategy by comprising a noble cause and socially beneficial components. According to this viewpoint, accountable administration is closely related to social affairs and substantially influences how society controls the collective welfare^64^. Consumer happiness is influenced by CSR programs that benefit both the company and its consumers and favorably influence communities and the quality of life for its constituents. Beyond benefiting society, making consumers happy helps businesses since it’s an excellent marketing approach that fosters emotional attachment and devotion^65^. Increased levels of CSR perception are connected with increased brand identity and consumer engagement. Still, this finding is conditional on the consumer’s ability to absorb and understand the information presented^66^. The severity by which consumers may be happy may depend on a variety of factors that touches on social media advertising content^67^. The following is how perception of a brand can influence the level of consumer happiness with life depending on the feelings towards brand experiences^68^. In this context, CSR activities of businesses enhance consumer emotions and can help affect positive change in consumer advocacy, consumer opinions and consumer happiness^69^.

**H1a**. corporate social responsibility positively influences consumer happiness.

Emotional connection is the bond formed when a consumer resonates positively with a specific brand, feeling affection, connection, or passion^70^. Consumers’ views on CSR typically build up positive moral standing and enhance a company’s unique assets, like trust and reputation^71^ that could lead to emotional attachment^47^Events at the workplace like corporate acts of corporate social responsibility can influence how consumers think and feel, such as their attachment to the company, which then leads to their behavioral reactions like willingness to pay. Therefore, we predict that the connection between CSR and their WTP will be influenced by emotional attachment. Individuals who view the company in a favorable light regarding its CSR initiatives are more likely to develop a strong emotional attachment to the business, leading to an increased likelihood of participating in willingness to pay.

**H1b.** CSR positively influences emotional attachment.

CSR plays a major role in boosting consumer retention through trust-building and creating emotional ties with consumers^72^. Engaging in CSR activities demonstrates a commitment to ethical conduct and societal concerns, effectively resonating with consumers. This ethical behavior promotes trust by giving consumers greater confidence and security in selecting a business that prioritizes social impact over profit. Engaging in CSR activities can improve connections with different stakeholders, leading to increased consumer retention^73^. Having trust is crucial because consumers who are loyal are more likely to continue purchasing from a business they trust and have an emotional bond with. Additionally, corporate social responsibility efforts frequently concentrate on issues that hold significance for consumers, nurturing a deeper emotional attachment that extends beyond business transaction. Observing a business support environmental sustainability or community development makes people feel proud and happy for supporting that business. This emotional attachment enhances their overall perception of the business, boosting the probability of their long-term retention. For instance^74^, found that effective CSR communication can boost business profits by enhancing business reputation and customer retention. By standing out in a competitive market and aligning with consumers’ values, CSR not only keeps existing consumers but also turns them into enthusiastic advocates, ultimately enhancing long-term consumer retention.

**H1c.** corporate social responsibility positively influences consumer retention.

These CSR aspects are impactful in determining willingness to pay for products and services among consumers. Businesses that align with this framework effectively are likely to elicit consumer’s positive affect and identification with these views, which translates into consumer’s devotion to brands as well as perceived value of offerings^75,76^. Through the societal marketing concept, consumers are happy to pay high costs for products or services from organizations that they feel are socially responsible and thus, portray strength of character that is in a position to work for the betterment of society^11^. However, it is also important to note that the overall changes in willingness to pay due to CSR are influenced by such factors as the characteristics of the specific CSR activities and consumer attributes such as age or gender and the perceived sincerity of the business’s actions^77,78^. Overall, CSR strategies that are in tune with consumer’s consciousness may align the business with an advantage and a higher consumer willingness to pay for its products or services.

**H1d**. CSR is positively associated with consumers’ WTP.

### Service quality, consumer happiness and willingness to pay

Today service has become a platform for the marketing companies to act in the interest of the consumers and make consumers happier mostly by providing better outcomes. Marketing goals should therefore be integrated with good service to ensure that consumers are greatly appealed and this creates a favorable and polished experience that keeps consumers loyal and with the passage of time the businesses gain competitive advantage and thus achieve their long term goals^79,80^. The goal of service marketing no longer just to satisfy the consumer’s need. It has expanded its perspective concerning the improvement of consumers happiness and their overall quality of life. This shift of thinking acknowledges that consumers are more than the utilitarian aspects of receiving service. It accepts the fact that there are few instances in life, which can immediately transform the mood and overall well-being of a person. Being a subcategory of the services marketing, the consumers’ happiness aims to make meaningful connections, strive to build long-term relationships, and provide the emotional appeal to the consumers^81,82^. The social marketing perspective believes that the application of marketing should also benefit consumers, or in other words, make them happier. Therefore, as per the social marketing concept, socially relevant aspects play the role of marketing outcomes^83^. Moreover, the notion of social marketing evaluates the social effect of service marketing on consumer happiness^84^.

Consumer satisfaction refers to the extent to which people feel they have experienced improvements to their general well-being and quality of life. Consumer satisfaction embraces individual judgments regarding prevailing living conditions^17,85^. Past studies have established^86^that a sequence of service encounters has a crucial impact on the perceptions that drive consumers’ judgments of their level of happiness. Additionally^87^, suggests that happy consumers are the result of their contentment with physical events spilling over into other areas of their lives. To put it another way, this study claims that combining CSR with service quality resulted in higher consumer happiness. Based on a survey of the pertinent literature, this study offers the arguments in favor of the positive relationship of CSR with consumer happiness.

**H2a**. service quality is positively associated with consumer happiness.

Positive emotions felt by consumers during service interactions are influenced greatly by service quality, especially intangible aspects such as personnel quality, outcome quality, and social quality^88^. These significant feelings then further strengthen consumer engagement, which is defined by a deep emotional connection and retention to the business^89^. Moreover, optimistic feelings indirectly enhance consumer advocacy, shown by consumer’s readiness to endorse and speak positively about the business because of their emotional attachment^88,90^. Physical aspects of service quality, indirectly improve emotional attachment by boosting perceptions of intangible factors like staff and social quality. In general, when business provide exceptional service in different areas, they can create positive feelings, leading to a strong emotional connection shown through increased engagement, retention, and advocacy actions^91^.

**H2b.** service quality is positively connected with emotional attachment.

The service quality is a critical driver of consumer retention through creating memorable and positive experiences. The provision of quality service consistently establishes the brand image with a sense of trust and reliability in consumers’ perceptions, thereby increasing the prospects of retention^92^. Provision of top-notch service often involves personalized care, timely response, and a thorough understanding of consumers’ needs, resulting in the establishment of emotional bonds and overall consumer satisfaction. For instance^93^ one such study conducted by [author] revealed a direct relationship between service quality and consumer satisfaction, which in turn leads to higher retention. This phenomenon not only instills a feeling of appreciation among consumers but also strengthens consumers’ loyalty towards the organization, encouraging them to promote the company to others, thus creating a productive retention and advocacy cycle.

**H2c.** service quality positively influences consumer retention.

Service quality is a key driver of consumers’ willingness to invest in a given service or product. Consumers, when they experience a higher level of service quality, are more willing to pay a premium price, associating superior service with greater value and satisfaction^94^. n the other hand, poor service quality can drastically reduce a consumer’s willingness to pay, as they might view the service as having a lower value compared to its price^95^. Key factors like reliability, responsiveness, empathy, and the physical aspects of service delivery are essential in contributing to the overall measure of quality. This measure, in turn, affects consumers’ perceived value and their willingness to pay premium prices for the service^94^.

**H2d**. Service quality is positively associated with consumers’ WTP.

### The mediating role consumer happiness

The evaluative result of CSR is happiness, which impacts behavioral outcomes in numerous ways. When consumers learn that a business is socially responsible and participates in CSR initiatives, they acquire a good impression of the company and a sense of retention^96,97^. analyzed at the influence of commitment and involvement as a mediating character in the connection of CSR and behavioral outcomes. According to previous research, business image, perceive quality and satisfaction influence brand loyalty^98,99^ which may lead WTP. Even though there has been no earlier research that explicitly looks at the connection of consumer happiness with WTP for CSR, existing studies imply that a happy consumers might pay more because of managerial execution, not really for corporate societal or ecological execution^100,101^.

**H3a**. consumer happiness has positive influence on consumers’ WTP.

Consumer happiness plays an important mediating role in CSR, service quality, and WTP (Willingness to Pay). By engaging in CSR activities and providing quality services, companies contribute to consumers’ well-being and subsequently gain satisfaction and emotional responses^8,66^. Happy consumers tend to develop greater brand loyalty and also a higher willingness to pay premium prices eventually benefiting the business^85^. Moreover, happiness can enhance the perceived value of both well-being and service quality, leading consumers to commit brand loyalty^102,103^ which may enhance WTP. Moreover, positively satisfied consumers conduct positive word-of-mouth and brand advocacy behavior and such positive association influences their support. Thus, organizations can take CSR and service quality to be at the forefront of consumer happiness which will, in turn, have a significant impact on WTP, thereby leading to sustainability of customer engagement and profitability.

**H3b.** consumer happiness has positive mediating influence on the relationship between CSR and WTP.

**H3c.** consumer happiness has positive mediating influence on the relationship between service quality and WTP.

### The mediating effect of emotional attachment

Gilal, F. G. et al., (2020)^104^ proposed that corporate social responsibility directly influences consumers’ affection and loyalty towards a business. People tend to identify with a company through sharing an affinity with its CSR activities and thus reinforcing their positive feelings towards their social identity^105^. When they identify an organization that is socially responsible, consumers feel satisfied while satisfying self-definitional and self-expression needs^106^. As a result of their high level of loyalty towards a brand, consumers are likely to adopt a defensive style of information processing, as this aligns with their expected outcomes. Companies that create a brand personality through CSR and establish an emotional connection with consumers actually position themselves to create sustainable relationships^107^. Even when faced with negative information, consumers who have built a deep emotional connection with an organization are likely to continue with the relationship.

Consumers’ feelings and thoughts about a specific business, connection to the business, and business visibility significantly impact their attitudes and actions^108^. Existing research has indicated that emotional connection with consumers not only enhances their extra-role actions, but also influences in-role behaviors like consumer cooperation, purchase intentions^109^. For instance, Cheng et al., (2016)^110^ study indicates that extra-role behaviors are influenced by consumers emotional attachment to a business through perceived value. Based on the earlier conversation, we anticipate that consumers who see or participate business’s CSR efforts will develop a stronger emotional attachment, leading to an increased willingness to pay. Thus, we formulated the following hypothesis.

**H4a.** emotional attachment positively influences consumer’s WTP.

As for emotional attachment, it served as a key mediator between CSR, service quality and WTP. When consumers believe a business is socially responsible and provides high-quality service, they have an emotional connection to the business that enhances trust in the business and commitment to it over the long term^111^. Such an attachment promotes loyalty and reduces price sensitivity, resulting in consumers’ willingness to pay premium for socially responsible business^71^. Research shows that emotional attachment towards business goes beyond just increasing positive word-of-mouth and coming back for repeat purchases, but it facilitates such behaviors even when negative claims are made or the cost of the brand increases^104^. Moreover, businesses that successfully align CSR with high service quality produce a feeling of belonging and identity with consumers, hence enhancing their readiness to contribute financially to the brand. Consequently, emotional attachment is an essential psychological tool that converts consumer’s investments in CSR and service quality into behavioral and financial commitment.

**H4b.** emotional attachment mediates the relationship between CSR and WTP.

**H4c.** emotional attachment mediates the relationship between service quality and WTP.

### The mediating role of consumer retention

Consumer retention is defined as “a profound commitment to continue promoting or making regular purchases of a selected good or service in the future, leading to repeated purchases of a similar business or service, regardless of contextual factors and advertising campaigns having the capability to trigger switching behavior”^112,113^.

The literature shows that satisfaction favors consumer loyalty^114^. For the formation of retention, this has been widely accepted that sustained satisfaction is necessary, at least in the beginning^115^. Consumer satisfaction are more likely to exhibit loyalty, which may be assessed behaviorally or attitudinally^116^. Positive, concrete behaviors like repurchasing products or services and referring to others are included in behavioral measures of consumer retention. On the other hand, attitude metrics reveal a business’s emotional and psychological ties. They entail dedication, involvement, and attitude towards the business. Price sensitivity has decreased, implying a larger price acceptance range. As a result, faithful consumers are more inclined to embrace a price rise to keep their registration or connect with their chosen service provider.

According to Rather and Sharma, (2016)^117^true loyalty might be illogical or prejudiced. This bias is defined as a halo influence, in which a consumer’s decent approach to a product has a spillover impact on their judgment of a particular aspect of a good or service. The sound effects of desired qualities, such as a company’s CSR activity, could be exaggerated. In contrast, the negative effects of undesirable qualities, such as a price increase, could be understated or ignored. Consumers may be prepared to pay for CSR if they have formed a strong feeling of loyalty or devotion to the service provider, as evidenced by the debate.

**H5a**. consumer retention is positively associated with consumers’ WTP.

Consumer retention can connect CSR and service quality with WTP. The research demonstrate that CSR and high service quality offered by businesses to consumers increase the level of trust and ability to form long-term ties with consumers, resulting in higher retention^118,119^. Retained consumers have a deeper commitment to business than competitors, an affinity that allows them to ask for an attitudinal premium even when their competition is substantially cheaper^93^. Research indicates that consumers feel more emotionally attached to business that engage in CSR activities, which leads to repeat purchase behavior. Thus, aligning CSR with high service quality builds retention, which reinforces consumers’ willingness to pay.

**H5b.** consumer retention mediates the relationship between CSR and WTP.

**H5c.** consumer retention mediates the relationship between service quality and WTP.

**Fig. 1:**
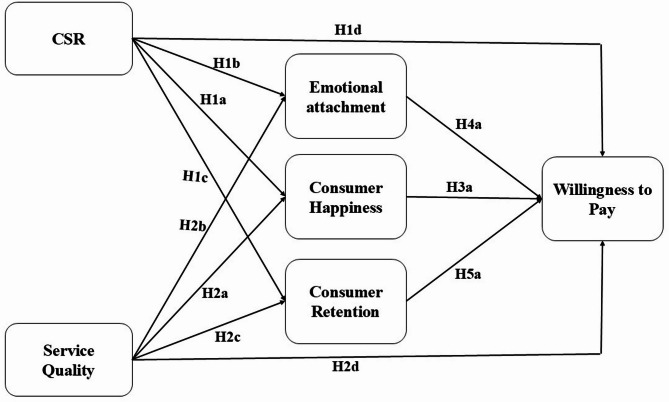
Conceptual framework.

As shown in Fig. 1, this study identifies and displays the model that will be empirically evaluated. All of the suggested hypotheses are included in the framework.

## Methodology

### Instrument development

For assessing the constructs, we employed multi-item measures previously used in research. A five-point Likert scale measured all components. A framework developed by^9,42^ was used to assess the five CSR elements. Four components of service quality were modified from^120^. Five items related to consumer happiness were taken from^62^. Four items for attitudes toward CSR were added from^121^. Four items were modified from^122^ for consumer retention. Three items were taken from^123,124^ for willingness to pay. The items and their resources are presented in Table 1. The XX Brand (anonymous) was selected for this study due to its strong CSR reputation, which is relevant to understanding how CSR affects consumer behavior. To ensure that brand preferences do not influence survey responses, we focused on assessing general perceptions of CSR, rather than personal attitudes toward a specific business. This approach mitigates bias and makes the findings applicable to other businesses with similar CSR practices. Many studies used such anonymous questionnaire^8,125^.

**Table 1:** Measurement items.

Construct	Items	Sources
CSR	The XX brand’s manufacturing process has sufficient labor hours. The XX brand is cruelty-free and does not engage in any practices that cause harm to animals. The XX brand enthusiastically engages in social welfare services initiatives. This XX brand works hard to increase its productivity. This XX brand aspires to be financially successful in the long run.	^42,119^
Service quality	The overall services offered as a whole are satisfactory. They are focused in their devotion and concern. They are very passionate about their work. They carefully consider our inputs and follow our recommendations thoroughly.	^118^
Consumer happiness	The XX CSR policy meets my professional standards. Due to XX commitment to socially responsible initiatives, I highly recommend them to others. It makes me delighted whenever I receive news about a CSR initiative. The CSR actions of company makes me happy. Overall, I like XX’s products and services.	^62^
Emotional attachment	I appreciate XX’s involvement in CSR initiatives. I have a feeling of fitting in when it comes to XX. I am pleased to be a user of XX. XX highly regards it’s all stakeholders, especially consumers.	^71^
Consumer retention	My first preference is the XX. I would suggest the services of the XX to others. I would recommend that others use XX’s services. Regarding the XX, I have only good things to say.	^122^
Willingness to pay	I am prepared to pay a premium to recognize the XX brand’s commitment to CSR. I will remain committed to XX brand for its CSR initiatives. I will continuously support the XX brand due to its CSR initiatives, if it charges higher than its competitors.	^123,124^

### Data collection

We chose to collect data in Pakistan using a paper questionnaire survey targeting Pakistani consumers since Pakistan is a developing country with increasing CSR awareness. A survey with 350 respondents from Pakistan was conducted to investigate the links between CSR, service quality and consumer willingness to pay (WTP). We selected Pakistan as the focus of this study. As a developing economy, there is an increasing awareness about CSR in Pakistan with the exposure of global business practices especially from global multinational companies. In this respect, it serves as an interesting case to study how CSR initiatives play in connection with consumers in a market moving towards increased social and environmental accountability.

The second reason is that Pakistan is facing socio-economic challenges including climate change, poverty, and poor working conditions, and CSR can play a significant role in circumstances like these as social concerns. These factors make Pakistan an ideal context in which to examine the impact of socially responsible business practices on consumer behavior, particularly in contexts where CSR is still in its infancy. Pakistan presents a highly relevant context for examining CSR and service quality due to its rapidly evolving market dynamics and increasing public sensitivity toward ethical business practices. The services sector contributes 58% to the Pakistan’s GDP^126^ with a rising emphasis on customer satisfaction and value-driven branding. CSR and sustainable initiatives in Pakistan have also gained momentum, as reflected in the Securities and Exchange Commission of Pakistan’s (SECP) CSR guidelines that encourage firms to report on social and environmental impacts^127,128^. These indicators collectively make Pakistan a compelling site for investigating the influence of CSR and service quality on willingness to pay. The educated urban consumers, who have the likelihood of knowing CSR and its impacts, were targeted to get valuable insights about the consumer perceptions and behavior. Data were gathered from the major urban areas of Islamabad, Peshawar, Lahore, Karachi, Faisalabad, Gujranwala, and Sialkot to establish a diverse group of respondents. Due to the lack of sample frames, databases, or random selection methods, convenience sampling was utilized within the cities to generate wider representation. While certain academics have criticized convenience sampling because of its capacity to produce outcomes that may not be applicable in the broader population, prior research suggests that the use of educated urban respondents can result in valid findings^129^. Educated urban consumers were targeted in the current study and were more likely to respond to the survey due to their greater awareness of CSR^130,131^. The procedure of data collection complied with the guidelines stated in the Declaration of Helsinki, and ethical clearance was obtained from the ethics committee of Shenzhen University. The survey instrument was divided into two sections: the first section reported demographic variables, namely, gender, age, education, and income. Data collectors approached several venues, such as colleges, educational institutions, government offices, supermarkets, restaurants, hotels, and corporate organizations, to receive responses.

The information was gathered during January to March of 2024. A total of 387 individuals responded to the 500 questionnaires that were delivered. To ensure the quality and reliability of the responses collected during the survey, the data cleaning process was conducted. Out of 387 responses obtained, 37 were excluded for completeness, inconsistencies, or invalid entries. It is an accepted practice to drop those responses in which over 10% of questions were left unanswered, and we followed that guideline here. Responding the same way (e.g., through selecting Strongly Agree in every single item) through multiple items in the whole survey was detected and dropped. Responses with outliers or inconsistent demographics (e.g., impossible ages or incomes) were flagged and excluded. After removing 37 incomplete responses, the data cleaning process yielded 350 valid surveys.

Data was collected through a non-probability convenience sampling technique involving 350 consumers in the urban centers of Pakistan. This method has been selected mainly because of the accessibility and its feasibility in terms of available time and budget, though a limitation of convenience sampling is the lack of generalizability, it is popular and valid in exploratory and theory-testing studies of the social sciences research^132^. Participants of different age groups and professions as well as different income groups were strived to be included in order to be more representative. Also, the number of participants in the sample was more than the minimum sample size drawn to use structural equation modeling (SEM), and thereby giving this study the appropriate statistic advantage in analysis.

In measurement and structural models, it is crucial that the regression sample size for the Partial Least Squares (PLS) path model be at least ten times larger than the number of independent variables^133,134^. Following guidelines stipulated by established research practices, the sample size required for the PLS path model needs to be ten times the greatest number of arrowheads pointing towards each latent variable. Based on this criterion, a sample size of 350 will be adequate. Table 2. confirms that there were 222 male respondents and 128 female respondents, with the majority of the respondents aged between 21 and 40. A large number of respondents held bachelor’s and master’s degrees. The 24 questions of the study used a five-point Likert scale that ranged from strongly disagree (1) to strongly agree (5). SmartPLS was used to validate the data and structural models, as supported by details in Table 2.

**Table 2:** Demographic profile (n = 350).

Total number of respondents		Frequency	Percentage
Gender	Male	222	63.42%
	Female	128	36.57%
Age	20 to 30 years	143	43.46%
	31 to 40 years	102	31.00%
	41 to 46 years	51	15.50%
	47 and above	33	10.03%
Education	Bachelor Degree	96	27.42%
	Diploma/Certificate	72	20.57%
	Postgraduate Degree	182	52.00%
Profession	Students	40	11.42%
	Entrepreneur	123	35.14%
	Employees	157	44.85%
	Retired etc.	30	8.57%
Income	0–35,001 RS	60	17.14%
	35,002 RS-45,001 RS	117	33.42%
	45,002 RS–51,001 RS	89	25.42%
	51,002 RS-75,001 RS	54	15.42%
	75,002 RS and above	30	9.57%

## Data analysis and results

### Methods

In the previous decades, covariance-based structural equation modeling (CB-SEM) was used extensively in analyzing complex relationships between latent and manifest variables. However, by 2010, the number of academic articles that applied partial least squares structural equation modeling (PLS-SEM) exceeded the number that used CB-SEM^135^. Recently, the publication rate using PLS-SEM has consistently outpaced that of CB-SEM. The PLS-SEM method has received significant appreciation among scholars due to its effectiveness in estimating models that have multiple constructs, structural paths, and indicator variables^134,136^. Another advantage of the method is its ability to estimate models without the need for specific assumptions about data distribution^137,138^. PLS-SEM refers to a form of structural equation modeling that analyzes inter relationships between latent variables by predicting causal constructs in statistical models. Many research studies have used PLS-SEM to analyze complex models with several constructs, paths, and predictors. Importantly, PLS-SEM does not require specific assumptions about data distribution^139,140^. This approach prioritizes prediction, modeling of specific interrelations, and estimation, and is classified under the general field of structural equation modeling^141^. The unique features of PLS-SEM provide a strong explanation, resolving possible trade-offs between explanatory power and predictive accuracy, which are necessary for gaining actionable knowledge relevant to management. In addition, access to user-friendly statistical software tools like SmartPLS and PLS-Graph increases the ease of access to these methods for use with limited technical expertise^142^.

PLS-SEM illustrates high expertise in evaluating complex models formulated from small sample sizes^143^. It presents efficient solutions in situations involving small sample sizes and models that consist of many elements and constructs. A recent study outlined^144^ how PLS-SEM can alleviate issues arising when alternative methodologies, including CB-SEM, do not converge using restricted samples and complex models. The authors posit that whenever a path model involves one or more constructs measured in a formative way, has a small sample size, incorporates several concepts, or faces issues related to normality of data distribution, PLS-SEM is the most beneficial approach. From this context, it can be concluded that PLS-SEM is highly suitable for the proposed study, especially owing to its exploratory nature and theoretical analysis focus. Data processing was performed using SmartPLS (version 3.2.1), as it is rated as more suitable compared to other structural equation modeling software^145^. In addition, it has the ability to calculate dynamic models that have various relationships^146^ particularly in situations where the estimates in the population are unclear^147^. PLS involves two combined models, an internal model that examines the relationships between latent variables and an external model that focuses on the relationship between latent variables and their respective indicators^148^. PLS eliminates the bias that arises in software covariance procedures, thus ensuring accuracy in results^133^. In evaluating the statistical significance of path coefficients, the bootstrapping routine was used^149^.

### Measurement model

The validity assessment of the reflective evaluation model depends on the internal consistency and the validity of the applied methodology. Theoretical hypotheses and conceptual framework shown in Fig. 1 were evaluated using Partial Least Squares Structural Equation Modeling (PLS-SEM). It is established that models that are highly complex can be efficiently analyzed using a smaller sample size. The PLS method imposes fewer restrictions compared to other modeling methods regarding sample size, data heterogeneity, and model complexity^148^. The data analysis was undertaken with the help of SmartPLS software, which facilitated the assessment of measurement precision and the establishment of relationships that are relevant to the study context. Confirmatory factor analysis was used to determine the validity and reliability of the measurement model by evaluating factor loadings, internal consistency, convergent validity, and discriminant validity. All items had factor loadings well above 0.7, thus implying highly reliable coefficients^150^. Internal consistency testing was performed through the application of Cronbach’s alpha, whose findings were above 0.7, thus confirming the reliability of the scale^151^. Convergent validity was confirmed by the fact that values of Average Variance Extracted (AVE) were above 0.5^155^. Based on the available literature^135^for the findings to be considered reliable, composite reliability (CR) should be above 0.7^157^. VIF values below 5 imply the non-existence of multicollinearity issues. These values are shown in Table 2.. The model’s goodness-of-fit was evaluated using the standardized root mean square residual (SRMR), with an acceptable limit of 0.08^158^. The SRMR value for the model was found to be 0.051, thus fulfilling the set criteria for acceptability^153^.

**Table 3:** Construct validity and reliability.

	Items	Loadings	VIF	Cronbach’s alpha	(rho_a)	(AVE)
CSR	CSR1	0.798	2.232	0.858	0.876	0.640
	CSR2	0.870	2.223			
	CSR3	0.829	1.945			
	CSR4	0.809	2.087			
	CSR5	0.783	2.398			
SQ	SQ1	0.897	2.626	0.841	0.861	0.680
	SQ2	0.875	2.275			
	SQ3	0.791	1.853			
	SQ4	0.724	1.489			
CH	CH1	0.731	1.307	0.789	0.701	0.514
	CH2	0.827	1.725			
	CH3	0.717	1.519			
	CH4	0.779	1.126			
EA	EA1	0.773	1.699	0.772	0.781	0.509
	EA2	0.796	1.169			
	EA3	0.750	1.822			
	EA4	0.717	1.213			
CR	CR1	0.813	1.131	0.715	0.833	0.504
	CR2	0.722	1.433			
	CR3	0.773	1.523			
	CR4	0.717	1.844			
WTP	WTP1	0.840	1.915	0.830	0.848	0.745
	WTP2	0.873	2.152			
	WTP3	0.875	1.773			

### Discriminant validity

Discriminant validity (DV) remains a concept that refers to a process of evaluating the distinctiveness of the measurements employed within a model from other aspects. DV has been assessed by prior research using various methodologies, cross-loadings and Fornell-Larcker criterion. Nevertheless, Henseler et al.^144^ detected a gap in the DV and noted that the identified criteria were not very sensitive. Therefore, the researchers introduced a new measure called Heterotrait-Monotrait Ratio (HTMT) to assess the discriminant validity of the constructs to offer a more accurate and sensitive method. he HTMT criterion suggests that the items which constitute the constructs are treated as non-replaceable whenever the correlation coefficients are less than the 0.90 threshold. Measuring this criterion is crucial in determining discriminant validity^144^. HTMT ratio was applied in the context of the proposed formulation, and the results are depicted in Table 4.. As shown from the results, the correlation coefficients for all the basic constructs are less than 0.90, thus indicating sufficient discriminant validity.

**Table 4:** Heterotrait-monotrait ratio (HTMT).

	EA	CR	CH	CSR	SQ	WTP
CR	0.654					
CH	0.812	0.455				
CSR	0.787	0.493	0.438			
SQ	0.874	0.483	0.574	0.345		
WTP	0.604	0.811	0.567	0.427	0.521	

### Structural model results

**Fig. 2:**
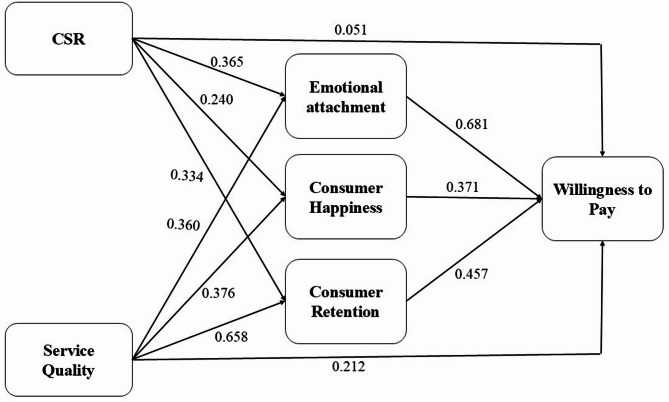
Structural model results.

The direction coefficients and R2 values for the research model that has been proposed are shown in Fig. 2. A determination of the significance of each route coefficient is made by analyzing the t-values that are produced by the PLS bootstrapping technique. Within the framework of the structural model, the results of the bootstrapping technique are presented in Table 5.. The findings suggest that eight of the hypotheses are correct, with the exception of the fourth hypothesis, which was shown to be rejected. (Fig. 2; Table 5.)

**Table 5:** Structural model results.

Hypotheses	Paths	β	*P* values	Results
H1a	CSR -> CH	0.240	0.000	Supported
H1b	CSR -> EA	0.365	0.000	Supported
H1c	CSR -> CR	0.334	0.000	Supported
H1d	CSR -> WTP	0.051	0.259	Not Supported
H2a	SQ -> CH	0.376	0.000	Supported
H2b	SQ -> EA	0.360	0.000	Supported
H2c	SQ -> CR	0.658	0.000	Supported
H2d	SQ -> WTP	0.212	0.000	Supported
H3a	CH -> WTP	0.376	0.000	Supported
H4a	EA -> WTP	0.681	0.000	Supported
H5a	CR -> WTP	0.457	0.000	Supported

### Path coefficient and confidence intervals

Furthermore, all major hypotheses were confirmed by the study. As per methodologies used in earlier research, a 95% confidence interval was generated using a bootstrap method using 5,000 resamples to determine the indirect effects^154^. Results of the mediation analysis highlighted the role of consumer retention and CSR perception as mediating variables in the relationship between CSR and WTP. Results of the PLS bootstrapping are shown in Table 6..

**Table 6:** PLS bootstrapping results.

Hypotheses	β	SD	T statistics	*P* values	Results
CSR -> CH	0.240	0.048	5.006	0.000	Supported
CSR -> EA	0.365	0.045	8.125	0.000	Supported
CSR -> CR	0.334	0.051	6.549	0.000	Supported
CSR -> WTP	0.051	0.045	1.128	0.259	Not Supported
SQ -> CH	0.376	0.045	8.343	0.000	Supported
SQ -> EA	0.360	0.046	7.781	0.000	Supported
SQ -> CR	0.658	0.027	24.213	0.000	Supported
SQ -> WTP	0.212	0.023	9.218	0.000	Supported
CH -> WTP	0.371	0.047	7.895	0.000	Supported
EA -> WTP	0.681	0.028	23.930	0.000	Supported
CR -> WTP	0.457	0.052	8.793	0.000	Supported

### Mediating effect

The testing of the mediating roles of consumer happiness, retention, and CSR perceptions in the framework of the relationship between CSR, service quality, and willingness to pay provides support for the presence of indirect influences. All the associations depicted in Table 7. are statistically significant (*p* < 0.05), with very large T statistics, thus providing solid support for the indirect effect of CSR and service quality on willingness to pay through the mediation of consumer happiness, retention, and emotional attachment.

**Table 7:** Mediating effect.

Hypotheses	Paths	β	S D	T statistics	*P* values
H3b	CSR -> CH -> WTP	0.287	0.041	7.049	0.000
H3c	SQ -> CH -> WTP	0.296	0.031	9.549	0.000
H4b	CSR-> EA -> WTP	0.312	0.035	8.917	0.000
H4c	SQ -> EA -> WTP	0.248	0.034	7.228	0.000
H5b	CSR -> CR -> WTP	0.245	0.029	8.409	0.000
H5c	SQ -> CR -> WTP	0.238	0.041	5.804	0.000

*R*^2^ is the proportion of an endogenous construct’s variability explained by its predictor constructs^155^. In this study, the pre-specified R² cut-off points (small, 0.25; medium, 0.50; large, 0.75) are used to classify effect sizes. Each exogenous construct’s effect size is calculated using SmartPLS software. F² values of 0.02, 0.15, and 0.35 are often interpreted as reflecting small, medium, and large effects on the exogenous latent variable, respectively^156^. The corresponding f² values are shown in Table 8. The determined effect sizes match those found in the literature, where Q² effect values of 0.02, 0.15, and 0.35 are referred to as small, medium, and large, respectively^157^. A model is considered to have predictive relevance when the Q² value is greater than zero. Table 8. summarizes the Q² values for the latent constructs covered in the model.

**Table 8:** Predictive relevance.

Latent Variables	*R* ^2^	Q^2^	F^2^
Consumer Happiness	0.356	0.180	
Emotional Attachment	0.330	0.167	
Consumer Retention	0.433	0.220	
Willingness to Pay	0.639	0.368	
CSR -> CH			0.234
CSR -> EA			0.358
CSR -> CR			0.324
CSR -> WTP			0.152
SQ -> CH			0.253
SQ -> EA			0.261
SQ -> CR			0.613
SQ -> WTP			0.230
CH -> WTP			0.362
EA -> WTP			0.538
CR -> WTP			0.431

## Results

CSR has been a significant issue for organizations; it can aid in the decision-making process for firms and their stakeholders. First, hypothesis 1 was accepted. The results demonstrated that CSR positively influence consumer happiness (H1), which are in line with previous studies conducted by^62,158^. The second hypothesis (H2), impact of CSR on emotional attachment was found accepted. Hypothesis 3 was accepted as there was a positive relationship between CSR and consumer retention. The fourth hypothesis was rejected, which assumes that CSR positively influences WTP (H2). The results showed no direct relation between CSR and WTP, corroborated by the previous findings of^159^. The above results show that consumer happiness plays mediating role between CSR and consumers’ WTP. Hypothesis 5 postulated that service quality positively influences consumer happiness and was supported. Hypothesis 6 was supported with the positive impact of service quality on emotional attachment. Hypothesis 7, which explored the influence of service quality on customer retention, was proven to be supported. In addition, our research confirmed a connection between service quality and willingness to pay, thereby providing support for Hypothesis 8. These results were obtained in the realm of CSR and align with studies conducted in different contexts^94,160,161^. The study findings indicate that consumer happiness, consumer retention, and emotional attachment mediate the relationship between service quality and consumers’ willingness to pay.

The research showed that the WTP is positively associated with consumer happiness (Hypothesis 9). Additionally, the current research demonstrated that emotional attachment is a legitimate measure of willingness to pay (Hypothesis 10) and Hypothesis 11 was supported as well, as there was a positive correlation between consumer retention and willingness to pay. The study found that CSR positively impacts consumer happiness, emotional attachment, and retention, consistent with the cognitive dissonance theory that suggests individuals strive to align their thoughts. This means that pleased consumers are motivated and hold favorable beliefs about CSR. These new findings enrich the CSR literature and are in congruence with previous research in different contexts^43,162–164^. Finally, consumer happiness, emotional attachment and consumer retention jointly described WTP for CSR and service quality. This research sheds novel information on the chain antecedents of WTP in the CSR context. It also determines that consumers might not keen to pay for CSR but use it as a service selection criterion. The prior research has demonstrated the positive impact of CSR on consumers’ willingness to pay premium, while our study found no direct relationship of CSR with consumer’s willingness to pay but it can be enhanced through different aforementioned mediating variables. The second important finding is the positive impact of service quality on consumers WTP and the mentioned mediating variables strengthened the relationship further. These are the interesting findings of the study that all the mediating variables enhance the relationship between CSR and service quality with consumer’s willingness to pay.

## Discussion

The findings affirm that CSR positively influences consumer happiness, aligning with prior research that highlights CSR’s role in enhancing consumer perceptions and emotional well-being^15^. However, the unique contribution of this study lies in uncovering the mediating mechanisms consumer happiness, emotional attachment, and consumer retention through which CSR indirectly influences WTP. By exploring these mediating pathways, the study provides a more nuanced understanding of how CSR impacts behavioral outcomes, extending the existing literature that primarily focuses on direct effects.

This research investigated how CSR activities together with service quality influences WTP by utilizing emotional and relationship-based mediators. The study investigates emotional attachment, consumer happiness and retention thus covering an essential research space in current literature. CSR activates its effect on willingness to pay through consumer emotional engagement and relationship development with consumers. The discoveries provide clearer perspectives about how CSR affects consumer purchasing behavior and they provide establishments with helpful approaches to design better CSR initiatives.

This study adds significantly to the existing literature on CSR by examining its effects on WTP in particular. Recent research has also addressed CSR as a factor influencing consumer decisions, with trust, emotional engagement, and perceived value as the main mediators of the CSR purchase intention relationship. Specifically, Deng and Xu (2017)^15^ emphasized that CSR activities stimulate trust and consumer-company identification, thus affecting consumer loyalty and purchase intentions. Similarly, Diallo et al. (2021)^16^ found that CSR activities improve customers’ perceptions of consumer WTP by enhancing brand value.

In fact, the essentiality of service quality in enhancing CSR outcomes is consistent with recent results in hospitality and retail domains. Alawamleh and Giacaman (2021)^165^ established that CSR boosts customer satisfaction and retention, which are critical determinants of consumer loyalty. Likewise, De Keyser and Lariviere (2014)^79^ underscored that high service quality induces positive emotional experiences that enhance customer-brand connections, resulting in an increased WTP. Our findings support these insights, suggesting that a high quality of service strengthens the mediating effects of happiness, retention, and emotional attachment, thereby reinforcing the CSR-WTP association. This relationship underscores the importance of combining CSR with excellent service to maximize consumer engagement and loyalty. This study also considered how each contextual factor can have significant implications for the findings. There was no direct link between CSR and WTP, indicating that consumers in developing economies like Pakistan perceive CSR as an added value factor rather than the primary driver of financial decisions. This finding highlights the importance of contextualizing CSR in developing economies, where consumers might prioritize other factors over CSR in their purchasing decisions. The results are in line with Kuokkanen and Sun (2024)^77^who found that sincerity and relevance were not only key influences on the effectiveness of CSR initiatives. Thus, CSR should be viewed as one of the value drivers, with emotional and psychological factors playing a more prominent role in mediating its effects on consumer behavior. In addition, the vital mediating variables emphasize the importance of companies establishing emotional and psychological bonds with consumers to lead CSR to actual performance. These findings should encourage further research on the interactions between CSR, service quality and consumer psychology in different cultural and industrial contexts. Future studies could explore how these mediating effects might vary across different socio-economic contexts and consumer demographics.

### Theoretical contributions

The findings extend the current literature by highlighting that the relationship between CSR and WTP is not direct but is instead mediated by emotional and psychological factors such as consumer happiness, retention and emotional attachment. Our results add to this understanding by demonstrating that the role of CSR in shaping WTP is indirect and channeled through consumer happiness, retention and emotional attachment. This mediation process has been underexplored in previous studies and presents a significant contribution to understanding how CSR activities influence consumer behavior in a more nuanced way. The study adds depth to the literature on CSR and consumer behavior by examining CSR initiatives and their effect on consumers WTP through mediating role of emotional attachment, consumer happiness and retention. While existing research has focused on a direct link between CSR and WTP, this study demonstrates the indirect and mediating role of psychological and emotional factors. The study extends the work of^15^on consumer-company identification, which was suggested to be a significant mediator in consumer responses to CSR, by providing insights into other mediating effects in the CSR-WTP framework, emphasizing the significant role of consumer happiness, retention and emotional attachment. Relatedly, another important theoretical contribution is the incorporation of service quality into the CSR-WTP framework. Though service quality has been extensively studied as a determinant of consumer satisfaction and loyalty, this research shows that it can enhance the impact of CSR on consumer choices. This suggests an implication of service quality such that the study distinguishes business operational competence can complement the effects of CSR by being able to enhance happiness, develop emotional attachment, and ensure retention. These findings help fill the gap in literature on CSR and service quality by providing new insights into their interaction in terms of consumer choices.

Moreover, this study makes a contextual contribution by examining consumers in Pakistan, a developing economy with unique socio-cultural and economic characteristics. The results imply that CSR initiatives are perceived and implemented in diverse ways based on culture or economic context and thus such study cannot be generalized globally but rather studied in a context-based manner to better understand both CSR theory and practice in concrete terms. Overall, this research contributes to the literature by providing a theoretical framework that integrates CSR, service quality, and WTP through mediators, offering a comprehensive explanation of consumer behavior. It challenges oversimplified models that assume direct effects of CSR on WTP and instead underscores the importance of emotional and relational pathways. Additionally, the findings advance cognitive consistency theory, stakeholder theory, and perceived value theory by demonstrating their relevance in explaining complex consumer decision-making processes.

### Managerial implications

The practical implications of this study are linked with its results. There is no direct link between CSR and WTP but it can enhance emotional attachment, consumer happiness and retention, which indirectly improve WTP. Businesses should not only focus on CSR initiatives but also ensure that these initiatives are integrated with high service quality to maximize their impact on consumer behavior. Effective communication of CSR activities, especially those related to the social and environmental problems in the community, can help create stronger emotional bonds with consumers As suggested by Banker et al., 2023 ^166^, the alignment of CSR activities with consumer values increases perceived relevance and effectiveness. Therefore, businesses should prioritize communicating their CSR initiatives in ways that resonate with their target audience’s values and beliefs. Businesses can improve both consumer happiness and WTP by integrating CSR with high service quality. Such strategies can not only enhance profitability but also play a role in sustainable business growth and societal well-being.

CSR activities are more effective if they are aligned with high service quality. This indicates that CSR must not be the only vehicle of financial returns, but a complement to operational excellence, which delivers better output. Firms can merge CSR activities such as community development or environmental sustainability efforts with greater service delivery to provide a powerful value proposition. Business should align CSR with high service quality to enhance consumer happiness, retention and emotional attachment for financial benefits. This is recognized as the distinctiveness of services connected with intangible nature and flexibility^167,168^. It implies that CSR disclosures, advertisements and documentation are essential for informing consumers and managing their expectations and opinions regarding a business’s CSR involvement. The findings show that when consumer’s expectations and perceptions of CSR are well managed, their level of happiness rises, which in turn creates WTP for CSR. Managers should ensure that CSR activities are well-publicized and in line with consumer values. For example, it is crucial for the marketing campaigns to reflect how CSR activities align to address local issues, like climate change or poverty alleviation, creating emotional attachment and loyalty among consumers. These strategies could assist businesses in establishing a strong brand image that consumers connect with ethical and socially responsible behavior. Moreover, the study also highlights the need to establish emotional and psychological bonds with consumers. Managers can accomplish this by designing CSR programs that invoke positive emotions and align with consumer’s expectations. For instance, using storytelling and involving consumers in CSR-related practices can foster emotional attachment, happiness and retention which can lead to increased WTP.

### Limitations

Despite providing important insights into the role of CSR in explaining consumer behaviors, this work has several limitations that should be duly considered. The study explores three mediators— emotional attachment, consumer happiness and retention, without examining other possible mediators or moderators. For instance, whether trust, consumer-company identification, or perceived CSR authenticity influence WTP could offer deeper insights into the mechanism. The data is cross-sectional in nature, preventing the derivation of causal inferences between CSR initiatives and consumer behavior, which constitutes another limitation. Longitudinal studies, which track consumer responses to CSR over an extended period, could offer a more comprehensive understanding of how consumers perceive and react to ongoing CSR activities. Such studies could reveal long-term changes in consumer behavior, trust, and loyalty. Moreover, the sample consisted largely of urban, educated consumers, which may have limited the viewpoint of rural or less-educated populations. This potential sample bias could reduce the generalizability of the results, particularly in regions where CSR awareness and attitudes may differ based on education levels, urbanization, and access to information. The limited focus on certain demographic segments warrants further investigation into how CSR is perceived by different groups within society. This oversight presents a gap in understanding the full spectrum of emotional and cognitive processes that may mediate the relationship between CSR and consumer behavior. While this context contributes significantly to understand the local dynamics and consumer perceptions of CSR, it may limit the generalizability of the findings to other geographic contexts or developed economies with different consumer behaviors and attitudes toward CSR. This context-specificity warrants caution when applying the results to more diverse or economically advanced settings.

### Future research direction

Based on the limitations and results of this study, several suggestions for future research are proposed. Future research may extend the framework by adding more mediators and moderators. For example, investigating other mediating factors such as perceived CSR authenticity, consumer trust, or brand equity can provide a broader understanding of how these factors affect WTP in CSR context. Additionally, exploring the role of demographic variables (e.g., income levels, education, and cultural orientations) as moderators could offer deeper insights into how different consumer segments respond to CSR activities.

Similarly, studies comparing the results across regions or sectors would also be valuable, as future research aims to establish the generalizability of these findings. For example, studying CSR effects in developed economies or industries with high competition (e.g., luxury goods or tech) may lead to more context-specific generalizations. These comparisons would help determine whether the findings from developing economies are applicable to markets with different levels of CSR awareness and consumer engagement.

More longitudinal studies are necessary to understand the temporal aspects of CSR’s impact on consumer behavior. For example, longitudinal research can examine whether sustained CSR efforts increase emotional attachment and consumer retention over time, thereby strengthening the long-term effects of CSR activities on WTP. Furthermore, future studies should emphasize the role of communication strategies in maximizing the impact of CSR. Investigating how storytelling, consumer engagement, or digital media campaigns influence can provide insights to future research. Understanding how effective communication strategies can enhance consumer perceptions and engagement with CSR activities would benefit organizations seeking to improve their CSR impact.

Finally, investigating the interrelationship of CSR and service quality in different cultural contexts may enhance our understanding of how these constructs interact to improve consumer loyalty and WTP. Future studies can explore how service quality and CSR combine in different cultural and economic environments, offering a more holistic view of how businesses can build long-term relationships with consumers through responsible business practices.

## Conclusion

This research adds to the body of CSR knowledge by expanding theoretical and managerial perspectives. The theoretical framework of this study views CSR as a characteristic that increases consumer happiness when combined with a business’s service quality. Drawing upon the perceived value, stakeholder and cognitive consistency theories, this research proposes that combining SQ with CSR can enhance consumer happiness. Additionally, the proposed chain of links adds to the current understanding of consumer behavior by revealing the leading and covering antecedents of WTP for CSR. This study emphasizes the importance of emotional attachment, consumer happiness and retention in connecting CSR to WTP. An individual’s CSR attitude is identified as a key factor that may enhance consumer WTP for CSR. Furthermore, the study explains why prior research on CSR and WTP for CSR may have yielded inconsistent findings. In other words, previous research may have overlooked essential mediators. Future studies should incorporate the mediating impact of other factors in their investigations to investigate the direct association of CSR with WTP. The findings of the theoretical model indicate that CSR can be perceived as a distinctive trait or attribute of a business, which consumers are likely to perceive positively and associate with. Effective implementation of CSR to achieve its goals can lead to emotional attachment, consumer happiness and retention, which is crucial for consumers to be willing to pay for CSR. In conclusion, businesses prioritizing CSR as a strategic initiative are more likely to attract and retain consumers, contributing to long-term success.

## Data Availability

Data can be made avalaible on request from Sohail Ahmad.
